# Normalization of activated partial thromboplastin time and diluted Russell’s viper venom time ratios can be safely omitted in the assessment of lupus anticoagulant

**DOI:** 10.1016/j.rpth.2026.103376

**Published:** 2026-02-02

**Authors:** Adrian Kimiaei, Jovan Antovic, Charlotte Gran

**Affiliations:** 1 Department of Clinical Chemistry, Karolinska University Hospital, Stockholm, Sweden; 2 Department of Molecular Medicine and Surgery, Karolinska Institute, Stockholm, Sweden

**Keywords:** antiphospholipid syndrome, Lupus coagulation inhibitor, partial thromboplastin time, reference standards, viper venoms

## Abstract

**Background:**

Normalization of lupus anticoagulant (LA) ratios is recommended by both the Clinical & Laboratory Standards Institute and the International Society of Thrombosis and Hemostasis to reduce inter-assay and intra-assay variation. This study evaluates if the normalization step can be safely omitted.

**Methods:**

This retrospective observational study evaluated the diagnostic performance of non-normalized LA ratios. Anonymized patient data from routine clinical workflows were divided into 2 datasets: one for establishing optimal non-normalized cut-off values (*n* = 440) and one for validation (*n* = 306). Clinical classification (positive/negative) in the first dataset served as the reference standard, enabling the establishment of optimal cut-off values for the non-normalized LA ratios using receiver operating characteristic calculations and Youden’s J statistic with bootstrapping. These optimal non-normalized cutoffs were then used on the validation set to compare the number of reclassifications when compared with normalized LA ratios.

**Results:**

Receiver operating characteristic analysis demonstrated high discriminative performance of non-normalized LA ratios for both dilute Russell’s viper venom time (area under the curve 0.9818; 95% CI, 0.9462-1) and activated partial thromboplastin time (area under the curve 0.8926; 95% CI, 0.8077-0.9775). Youden’s J identified optimal cut-off values of 1.304 for dilute Russell’s viper venom time (95% CI, 1.297-1.307) and of 1.554 for activated partial thromboplastin time (95% CI, 1.504-1.562). Applying the above criteria to the validation dataset resulted in no reclassifications.

**Conclusion:**

The normalization step in the analysis of LA ratios can be safely omitted, allowing for a reduced laboratory workload, without affecting result interpretation and thus clinical outcomes.

## Essentials

• Laboratories adjust lupus anticoagulant tests to reduce variation.
• We studied large amounts of hospital data to test if this adjustment can be simplified.
• The new approach gave results identical to the current standard.
• Simplifying the process does not affect diagnosis and might further lessen the variation.

## 1 Introduction

The analysis of lupus anticoagulant (LA), one of the 3 laboratory criteria for diagnosing antiphospholipid syndrome, is based on ratio comparison between phospholipid-poor and phospholipid-rich reagents in LA-sensitive coagulation assays [1]. Current guidelines from the International Society on Thrombosis and Haemostasis (ISTH) advocate for a parallel analysis of the dilute Russell’s viper venom time (dRVVT) and the activated partial thromboplastin time (aPTT) [2] for LA detection. While these are the most robust methods available, they have significant limitations, including reagent variability, complex workflows, and susceptibility to interferences. Notably, there is a lack of reagent standardization, with significant variability between both manufacturers and reagent lots, calling for a normalization of results. Additionally, the multistep analysis process is cumbersome, adherence to guidelines is inconsistent [3] and the results are influenced by factors such as inflammation, anticoagulant therapy, and other preanalytical variables [4,5].

In addition to the ISTH guidelines, the Clinical and Laboratory Standards Institute (CLSI) has published separate guidelines for the analysis of LA [6]. While largely in agreement with ISTH, the CLSI guidelines differ in several recommendations, one of them being regarding the normalization of LA ratios. The CLSI guidelines propose normalization by dividing the patient test result by the mean of the reference interval [6], whereas the ISTH advises normalization by division of patient results with pooled normal plasma (PNP) measured in the same run [2].

Normalization offers several advantages: reduced lot-to-lot and inter-day variability [7,8], standardization between laboratories [9] and has in one study been associated with increased sensitivity in dRVVT testing [8]. However, both normalization methods retain uncertainties: PNP normalization is affected by manufacturer and lot variability, while reference interval normalization does not account for day-to-day analytical variation [10]. Furthermore, the normalization process is in practice nonstandardized and can increase laboratory workload. These issues are highlighted by a pre-guideline issuance survey performed by ISTH, showing disparities between laboratories regarding the normalization methods used in practice, with respondents using either the CLSI or the ISTH method and with PNP sources varying widely [3]. Given these challenges, it may be valuable to evaluate whether normalization in LA analysis can be safely omitted without compromising diagnostic accuracy.

## 2 Materials and Methods

### 2.1 Patients

The study was performed as a retrospective, observational study utilizing 2 separate datasets: one for training and one for validation. The training dataset was comprised of anonymized patient data from routine LA analyses performed at the Department of Clinical Chemistry at the Karolinska University Hospital, from December 2021 to January 2022. All analyses conducted during this period were included, excluding those who did not have a numerical result for both screening aPTT and dRVVT. CRYOcheck pooled normal plasma (frozen; Precision BioLogic) control values, used for normalization, for all 4 analyses (aPTT screen and confirm and dRVVT screen and confirm) were collected from January 2021 to January 2022. For validation a previously collected dataset from 2016 was used. This dataset included healthy controls, recruited from laboratory staff who self-identified as healthy and were not taking any antiplatelet or anticoagulant medication, and anonymized patients with positive LA, obtained from clinical routine LA testing using an identical methodology to the training dataset. In both datasets, the following data were collected: raw clotting times for the screening aPTT and dRVVT, PNP values for the concurrent run (used for normalization), and where applicable also values (raw clotting times for patients and PNP) for the confirmation tests.

### 2.2 LA analysis

Samples were treated according to clinical routine: drawn in 3.2% sodium citrate tubes, double centrifuged at 2000 g for 15 minutes at 15 °C within one hour from sampling and immediately aliquoted and frozen at −70 °C. Samples were thawed in a 37 °C water bath for 5 minutes and thoroughly mixed before analysis. Analysis was done on the Siemens BCS XP with LA1 screen and LA2 confirm for dRVVT, Dade Actin FS for aPTT confirmation (all Siemens) and PTT-LA (Stago) for aPTT screening. The 3-step procedure was followed, with mixing performed when both screen and confirm clotting times were prolonged, according to CLSI H60 [6]. LA positivity was assessed according to ISTH guidelines [2] with locally derived cutoffs. Patients treated with direct oral anticoagulants or vitamin K antagonists were pretreated before analysis with direct oral anticoagulants-Stop [11] (Haematex) or PNP mixing respectively, according to ISTH guidelines [2] and locally validated routines.

### 2.3 Statistics

Patients from the training dataset with available aPTT and/or dRVVT ratios (screen/confirm) were selected. Receiver operating characteristic (ROC) area under the curve (AUC) calculations were then used to assess the discriminatory performance of non-normalized (screen clotting time/confirm clotting time) LA ratios for both aPTT and dRVVT, using the classification (positive/negative) from the clinically utilized normalized (normalized screen ratio/normalized confirm ratio) LA ratios as the reference method. Patients in the training dataset with normalized screen clotting times below the locally established cutoff were classified as LA-negative and thus did not undergo confirm testing; consequently, they did not contribute to the ROC analyses. Confidence intervals for the ROC AUC were calculated according to DeLong et al. [12].

Cutoffs for the non-normalized ratios of aPTT and dRVVT were derived from the training dataset. The optimal threshold, corresponding to the highest Youden’s J, was identified through ROC analysis, and its distribution was estimated using 2000 bootstrap replications. The final cutoff was defined as the median of these bootstrap-derived thresholds, with a 95% confidence interval based on the percentiles of the bootstrap distribution. These new cutoffs were then applied to the validation dataset to evaluate the number of reclassifications.

Calculations and figures were done in RStudio (version 2023.06.1) using R (version 4.4.1). ROC analyses were performed with the pROC package [13].

## 3 Results and Discussion

A total of 440 patients were included in the training dataset. Among them, 70 (15.9%) had a normalized dRVVT ratio, and 55 (12.5%) had a normalized aPTT ratio. Both ratios were available for 35 patients (8.0%), while 350 patients (80.0%) had no normalized LA ratio available. In total 64 patients (14.5%) were positive using clinical routine cutoffs (with normalization). The validation dataset contained 49 healthy controls, of which all were LA-negative and had both aPTT and dRVVT ratios available, and 257 positive patients from the clinical workflow, of which 231 (89.8%) had a dRVVT ratio and 152 (59.1%) an aPTT ratio available.

**Figure 1:**
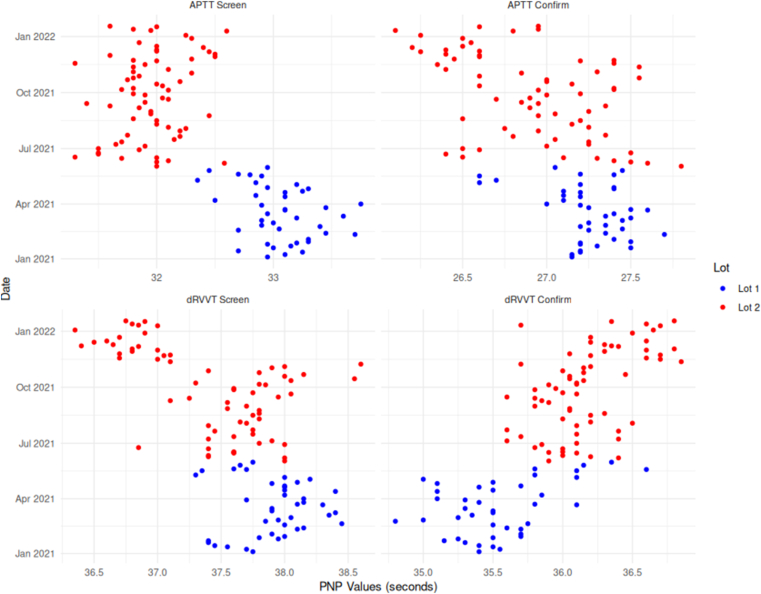
Scatter plot showing values for dRVVT and aPTT, screen and confirm, for different PNP lots. aPTT, activated partial thromboplastin time; dRVVT, diluted Russell’s viper venom time.

The PNP values for screen and confirm for both aPTT and dRVVT were used for normalization in the training dataset. In general, PNP showed a rather low variability with a %CV of 1.35 (dRVVT screen), 1.27 (dRVVT confirm), 1.89 (aPTT screen), and 1.42 (aPTT confirm). However, during the study period two lots of PNP were used, with a switch in June 2021 (Figure 1). This yielded relative percent differences of −3.4% (aPTT screen), −1.2% (aPTT confirm), −1.3% (dRVVT screen) and 1.7% (dRVVT confirm), aligning with one of the issues raised by Moore [10]. Moreover, Cabo et al. [14] recently demonstrated that variability between PNP lots can alter the cut-off values for normalized dRVVT ratios and thus positivity rates, further underscoring the limitations of PNP-based normalization, unless cut-offs are checked with each new PNP batch, which is time and resource expensive.

In our case, the lot switch led to a decrease of aPTT ratios by more than 2 percent, introducing a systematic bias based on the PNP lot. The issue could be further exacerbated by the day-to-day variability of the PNP measurement, where days with low PNP (by virtue of the variability described above) would unduly elevate the LA ratios and vice versa.

**Figure 2:**
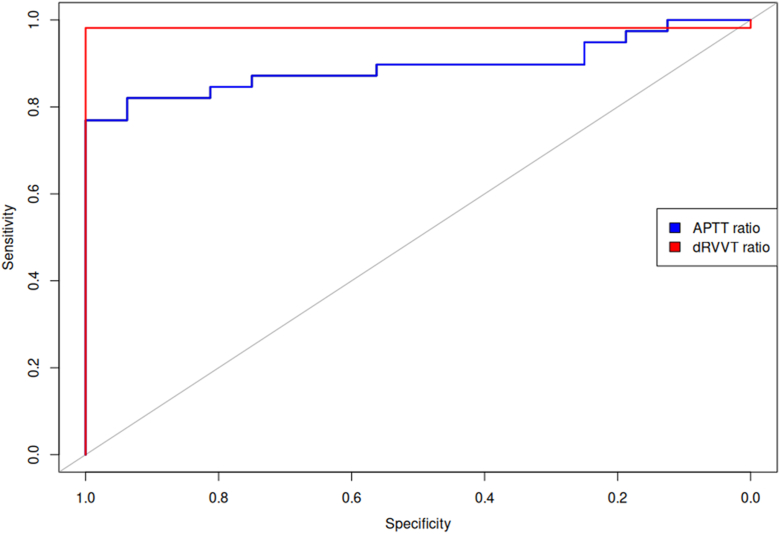
ROC plot showing the discriminatory ability of non-normalized ratios with the normalized ratios as reference standard. The dRVVT AUC is 0.9818 (95% CI 0.9462 – 1) and the aPTT AUC is 0.8926 (95% CI 0.8077 – 0.9775). ROC, receiver operating characteristic; AUC, area under the curve; aPTT, activated partial prothrombin time; dRVVT, diluted Russell’s viper venom time.

ROC AUC calculations demonstrated high discriminatory performance of non-normalized LA ratios, with a dRVVT AUC of 0.9818 (95% CI, 0.9462-1) and aPTT AUC of 0.8926 (95% CI, 0.8077-0.9775) (Figure 2). The median optimal cutoff (based on Youden’s J) from the bootstrap distribution was 1.304 for dRVVT (95% CI, 1.297-1.307) and 1.554 for aPTT (95% CI, 1.504-1.562). Compared to the cutoff limits for normalized LA ratios, the aPTT cutoff was proportionally higher, reflecting the inherent tendency of the aPTT confirm reagent to produce shorter clotting times than the aPTT screen, in contrast to the dRVVT reagents. Application of these new cutoff limits to the validation dataset resulted in no reclassifications, reinforcing that non-normalized LA ratios provide equivalent discriminatory performance to normalized ones.

While non-normalized LA ratios preserve the discriminatory performance of normalized LA ratios, variability remains a concern—both in day-to-day measurements and across reagent lots. In our data the day-to-day variability in %CV was comparable to the relative percent difference between the PNP lots, with the first being at random and the second one representing a systematic shift. Moreover, if daily normalization uses a single PNP measurement rather than an average of multiple measurements, there is a risk of selecting a distributional extreme. This could introduce a systematic bias affecting samples processed that day, with additional skew for patient samples that, by chance, fall on the opposite end of the distribution. Furthermore, normalization by dividing the analysis result with the control value is not a common practice, neither for other coagulation assays nor in the wider clinical laboratory.

Variation between lots is a larger issue and has been recognized for nearly half a century [15,16]. It is evident that compensation is needed, and we identify 2 potential solutions—both require evaluation of the new lot against the old one. Depending on manufacturer availability, the new lots can either be rejected until a suitable match is found, or a correction factor can be applied to align the new values with the previous lot.

**Figure 3:**
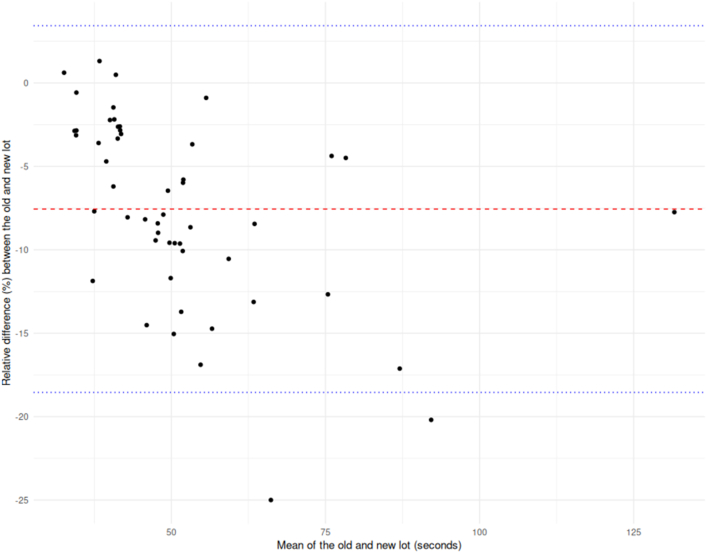
Bland-Altman plot comparing two reagent lots for the dilute Russell’s viper venom time screen (LA1, Siemens) across 52 samples. The y-axis represents the relative difference (%) between the lots, while the x-axis shows the mean of the two lot values. The red dashed line indicates the mean relative difference, and the blue dotted lines denote the 95% limits of agreement.

An interesting observation from our lot comparisons is that the relative difference between lots is not necessarily uniform across the measuring interval (Figure 3). In this case the observation is for the dRVVT screen reagent; however, similar effects cannot be excluded for other reagents. Since factorization applies a single correction value, it risks introducing bias at both the low and high extremes. However, the implications for normalization are even more problematic—if a new lot increases PNP by only 4% but elevates some patient samples by up to 15%, as seen in our data, all normalized LA ratios will be increased by approximately 10.6%. The only definite solution would be derivation of new cutoffs for each reagent lot as suggested by CLSI [6]; however, this is not feasible for the majority of laboratories. Since lot-based drift remains a concern regardless of whether normalization or factorization is applied, aliquoting the samples used to establish or verify locally applied cutoff limits and using them to monitor drift over time could serve as an alternative, provided that storage conditions are validated to maintain sample integrity.

Notably, one study suggested that normalization increases sensitivity for weakly positive LA [8]. The study used seven lyophilized samples (6 positive and 1 negative and heparin-treated), sent to 29 laboratories, all using a common author-supplied cut-off. In such an inter-laboratory scenario, normalization might indeed improve results. However, we do not see this in our data and their conclusion may not generalize to laboratories working with their own reference intervals. Another study reported discrepancies in LA positivity when comparing normalization with PNP and reference interval, especially for the aPTT ratio [17], but did not assess non-normalized LA ratios.

One argument in favor of normalization is its potential to reduce inter-laboratory variation [2,9], especially for laboratory networks using similar instrumentation and cut-offs. However, for the results to be truly comparable, assurance needs to be made that the same normalization procedure is utilized, preferably using the same PNP lot, which might not be feasible in practice.

Our study has several strengths: the use of a large, retrospective real-world patient dataset, division into separate training and validation cohorts and robust analysis methods such as ROC with bootstrapping. Due to the inclusion of a large, unselected LA-positive cohort, the study encompassed a continuum of weak, moderate, and strong LA across the analytical range, with a positively skewed LA ratio distribution as expected. Nonetheless, it also has limitations – notably, both the reagent combination and the mixing procedure used can influence LA ratio distributions and potentially affect the detection of weakly positive LA. Our approach to mixing, based on the CLSI H60 guideline, already aligns with the latest ISTH update [18], where samples with prolonged screen and confirm but a correcting mix are reported as LA-negative. Although this practice may have a dilutive effect on weak antibodies, no reclassification of weakly positive LA was observed in our study, indicating that this effect is not inherently related to whether LA ratios are normalized or non-normalized.

A further limitation is the lack of availability of clinical data due to the retrospective laboratory study design, which precludes assessment of APS status and therefore limits evaluation of clinical specificity. In addition, the aPTT reagents used in this study are not paired reagents as intended by the ISTH guidelines, as they differ in more than phospholipid concentration. Consequently, the aPTT screen/confirm ratio is not a pure indicator of phospholipid dependence, which may affect diagnostic specificity. However, it does not influence the primary aim of the study, which was to compare normalized and non-normalized analytical approaches using empirically derived cut-offs.

In summary, we show that the normalization step can be safely omitted in LA analysis. We see both benefits and drawbacks with removing normalization, and acknowledge that its feasibility may be greater for larger laboratories capable of establishing and effectively monitoring their own cutoff limits.

## 4 Conclusion

Non-normalized cut-off values for assessing positive LA ratios could be safely used on a validation dataset of over 300 research subjects, leading to zero reclassifications. This approach necessitates alternative strategies for managing lot-to-lot variability and may not be suitable for all laboratories. Nevertheless, our results support a different analytical process from the current guidelines and could be used to inform and provide alternatives to future editions.
